# 1-O-Hexyl-2,3,5-Trimethylhydroquinone Ameliorates the Development of Preeclampsia through Suppression of Oxidative Stress and Endothelial Cell Apoptosis

**DOI:** 10.1155/2021/8839394

**Published:** 2021-01-15

**Authors:** Lai Jiang, Yanping Gong, Jie Rao, Qiuhong Yang, Na Gao, Guiyang Li, Yuyan Ma

**Affiliations:** ^1^Department of Obstetrics and Gynecology, Qilu Hospital, Cheeloo College of Medicine, Shangdong University, Jinan, Shangdong 250012, China; ^2^Department of Obstetrics and Gynecology, The First Affiliated Hospital of USTC, Division of Life Sciences and Medicine, University of Science and Technology of China, Hefei, Anhui 230001, China; ^3^Department of Echocardiography, The First Affiliated Hospital of USTC, Division of Life Sciences and Medicine, University of Science and Technology of China, Hefei, Anhui 230001, China; ^4^Department of Obstetrics and Gynecology, Jinan Maternity and Child Care Hospital, Jinan, Shandong 250001, China

## Abstract

1-O-Hexyl-2,3,5-trimethylhydroquinone (HTHQ), a potent nuclear factor-E2-related factor 2 (Nrf2) activator, has potent antioxidant activity by scavenging reactive oxygen species (ROS). However, the role of HTHQ on the development of preeclampsia (PE) and the underlying mechanisms have barely been explored. In the present study, PE model was induced by adenovirus-mediated overexpression of soluble fms-like tyrosine kinase 1 (sFlt-1) in pregnant mice. The results showed that HTHQ treatment significantly relieved the high systolic blood pressure (SBP) and proteinuria and increased the fetal weight and fetal weight/placenta weight in preeclamptic mice. Furthermore, we found that HTHQ treatment significantly decreased soluble endoglin (sEng), endothelin-1 (ET-1), and activin A and restored vascular endothelial growth factor (VEGF) in preeclamptic mice. In addition, HTHQ treatment inhibited oxidative stress and endothelial cell apoptosis by increasing the levels of Nrf2 and its downstream haemoxygenase-1 (HO-1) protein. In line with the data in vivo, we discovered that HTHQ treatment attenuated oxidative stress and cell apoptosis in human umbilical vein endothelial cells (HUVECs) following hypoxia and reperfusion (H/R), and the HTHQ-mediated protection was lost after transfected with siNrf2. In conclusion, these results suggested that HTHQ ameliorates the development of preeclampsia through suppression of oxidative stress and endothelial cell apoptosis.

## 1. Introduction

Preeclampsia (PE) is a serious complication of pregnancy, which is one of the important causes of morbidity and mortality of pregnant women and perinatal infants. It refers to a group of clinical syndromes with hypertension and proteinuria as the main clinical manifestations after 20 weeks of pregnancy [1–4]. PE adversely affects maternal health and leads to substantial complications, such as eclampsia, ischemic heart disease, renal failure, liver injury, central nervous system injury, stroke, pulmonary edema, respiratory distress syndrome, and other complications. It also increases the risks of fetus growth restriction, placental abruption, prematurity, and even perinatal mortality, all of which negatively mediate fetal health [5–7]. Clinical data revealed that PE affects roughly 5% to 7% of pregnant women worldwide and led to the deaths of more than 70,000 maternal and 50,0000 fetal every year [8]. Therefore, great attention has been addressed to the identification of novel therapeutic targets and agents against the maternal syndrome of PE.

Although the pathophysiology of PE remains is not wholly clear, it is known that oxidative stress plays a pivotal role of PE development. Oxidative stress is characterized by the imbalance between the generation of reactive oxygen species (ROS) and the antioxidant defence system, [9, 10]. ROS can induce placental dysfunction by suppression of placental angiogenesis, induction endothelial damage, and immune malfunction, which are suggested to be the underlying of PE development [11–13]. Several studies have found suppressing oxidative stress, and scavenging ROS represents potential therapeutic opportunities for the treatment of PE development [11] [14].

1-O-Hexyl-2,3,5-trimethylhydroquinone (HTHQ), a derivative of vitamin E, has potent antioxidant activity by directly reacting with ROS and scavenging them to form more stable free radicals [15]. HTHQ is a potent nuclear factor-E2-related factor 2 (Nrf2) activator and induces the haemoxygenase-1 (HO-1) expression [16, 17]. In addition, HTHQ has positive effects on various diseases and conditions, including diabetes [18], hepatic cirrhosis [19], neurodegenerative diseases [20], and cancer [21, 22]. Previous researches showed that Nrf2/HO-1 pathway plays an important role in the development of PE [23, 24]. However, the effects of HTHQ on PE development remain unclear. Hence, the aim of this study was to investigate the effects of HTHQ on PE development as well as the associated mechanisms.

## 2. Materials and Methods

### 2.1. Animal and Model Establishment

Female (8-10 weeks) and male (for mating purposes only) C57BL/6 mice were purchased from Beijing Vital River Laboratory Animal Technology Co., Ltd. (Beijing, China). All mice were housed with a controlled environment and maintained in 12-hour light and dark cycles. Virgin female mice were mated with males, and the next morning, a white or faint yellow vaginal plug was detected in the 0.5 embryonic day (E0.5). The PE model was prepared by injection with 3 × 10^9^ PFU/100 *μ*L of adenovirus to overexpress sFlt-1 (Ad-sFlt-1) through the tail vein at days 7 to 8 of gestation as described in a previous study [25]. The mice in the control group were injected with 3 × 10^9^ PFU/100 *μ*L of adenovirus encoding murine Fc protein (Ad -Fc). In PE group, the mice were orally administered with HTHQ (100 or 200 mg/kg) every day from days E0.5 to E17.5 of pregnancy. The systolic blood pressure (SBP) was measured by noninvasive blood pressure analysis system and repeated at least three times. Albumin and creatinine levels were determined following the manufacturer's instructions on E17.5. All animal care and experimental procedures were conducted in strict according to the National Institutes of Health Guide for the Care and Use of Laboratory Animals and approved by the animal ethics and welfare committee of Anhui Medical University.

### 2.2. Plasma and Tissue Assays

On day 17.5 of pregnancy, the animals were euthanized, and peripheral blood was collected for further analysis. The mice were dissected; fetal and placentas were obtained to evaluate the weight. The circulating levels of soluble endoglin (sEng), vascular endothelial growth factor (VEGF), endothelin-1 (ET-1), and activin A were measured using enzyme-linked immunosorbant assay (ELISA) kits according to the manufacturer's instructions.

### 2.3. TUNEL Staining

Placenta tissues were fixed by perfusion with 10% formalin and embedded in paraffin and then cut into 4 *μ*m slides. Apoptotic cells were measured using a terminal deoxynucleotidyl transferase dUTP nick-end labeling (TUNEL) detection kit. All images were analyzed using a quantitative digital image analysis system.

### 2.4. Cell Culture and Treatment

Human umbilical vein endothelial cells (HUVECs) were purchased and cultured in Medium 199 supplemented with 20% fetal bovine serum, 1% antibiotics, and L-glutamine at 37°C under 5% CO_2_ and 100% humidity [26]. To knockdown the genes, the cells were transfected with si-Nrf2 using Lipofectamine 2000 according to the protocol recommended. After transfection with siRNA for 18 h, the HUVECs were treated with HTHQ for 6 h before hypoxia and reperfusion (H/R) insult. Briefly, the cells were cultured in preconditioned hypoxic medium under 5% CO2 and 95% N2 in a humidified chamber for 8 h and then replaced with fresh medium, and the cells were cultured under normal growth conditions for 16 h of reoxygenation.

### 2.5. Oxidative Stress Detection

Dihydroethidium (DHE) staining was preformed to assess the ROS production as described in a previous study [27]. In addition, placenta tissues and HUVEC concentrations of superoxide dismutase (SOD), catalase (CAT) malondialdehyde (MDA), and glutathione (GSH) were measured using commercially available kits.

### 2.6. Western Blotting

The total proteins were separated on SDS-PAGE gel, blotted onto PVDF membrane, and then blocked with 5% nonfat milk. Subsequently, the membranes were incubated with antibodies Bax (1 : 1000, Abcam), Bcl-2 (1 : 1000, Abcam), Nrf2 (1 : 1000, Abcam), HO-1 (1 : 1000, Abcam), and *β*-actin (1 : 1000, Abcam). The membranes were incubated goat anti-rabbit IgG secondary antibody (LI-COR) and analyzed by a two-color infrared imaging system (Odyssey; LICOR) to quantify protein expression.

### 2.7. Statistical Analysis

The data are presented as the means ± standard deviation (SD). Statistical differences between 2 groups were determined by Student's *t*-test. Statistical comparisons among multiple groups were compared by one-way analysis of variance (ANOVA) tests with a post hoc Tukey test. *P* values less than 0.05 were considered to indicate a statistically significant.

## 3. Results

### 3.1. HTHQ Treatment Ameliorated PE Development In Vivo

**Figure 1:**
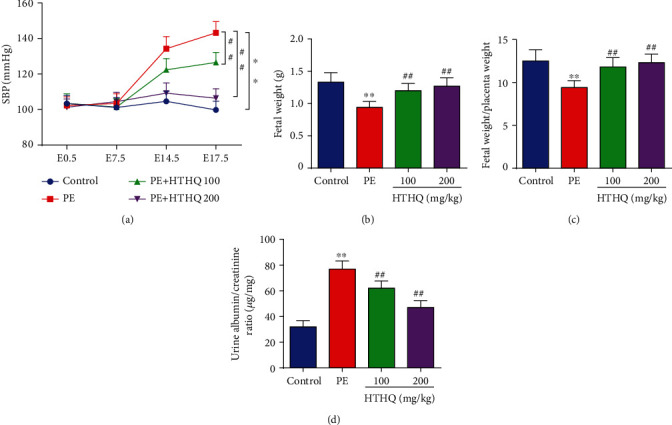
HTHQ treatment ameliorated PE development in vivo. (a) The systolic blood pressure (SBP) of mice was detected in each group (*n* = 8). (b) The fetal weight of mice was detected in each group (*n* = 8). (c) The fetal weight/placenta weight ratio of mice was detected in each group (*n* = 8). (d) The proteinuria of mice was detected in each group (*n* = 8). ^∗∗^*P* < 0.01 vs. the control group; ^#^*P* < 0.05 and ^##^*P* < 0.01 vs. the PE group.

**Figure 2:**
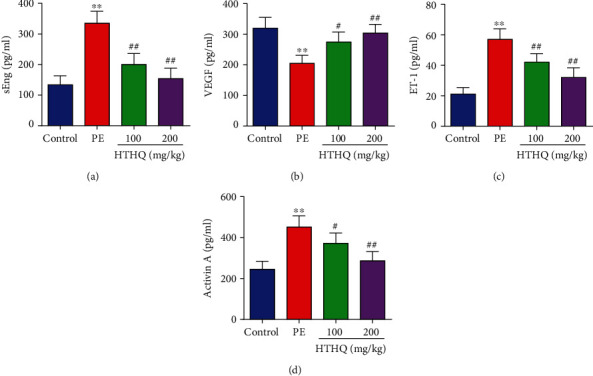
HTHQ treatment attenuated decreases the concentration of sEng, ET-1, and activin A levels and restored VEGF levels in blood from PE animals. ELISA was used to detect the concentration of sEng (a), VEGF (b), ET-1 (c), and activin A (d) in serum of mice in each group (*n* = 6). ^∗∗^*P* < 0.01 vs. the control group; ^#^*P* < 0.05 and ^##^*P* < 0.01 vs. the PE group.

As shown in Figure 1, the PE group has increased SBP and the proteinuria and decreased the fetal weight and fetal weight/placenta weight compared with the control group, indicative of successful model establishment. We further found that HTHQ treatment significantly relieved the high SBP and proteinuria and increased the fetal weight and the fetal weight and fetal weight/placenta weight in a dose-dependent manner (Figure 1). Notably, HTHQ treatment significantly decreased sEng, ET-1, and activin A levels and restored VEGF concentrations compared with the PE group (Figure 2), suggested that HTHQ treatment is able to relieve the symptoms of PE.

### 3.2. HTHQ Treatment Attenuated Oxidative Stress Induced by PE

**Figure 3:**
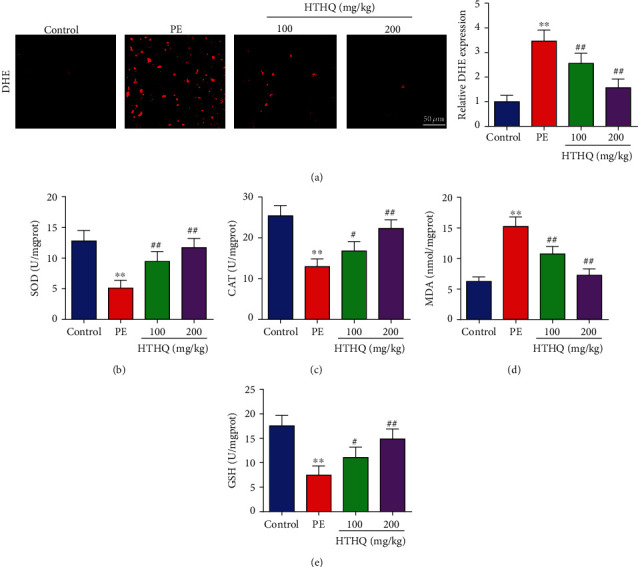
HTHQ treatment attenuated oxidative stress induced by PE. DHE staining was used to assess the ROS production in each group (*n* = 6). The levels of SOD (b), CAT (c), MDA (d), and GSH (e) in each group (*n* = 6). ^∗∗^*P* < 0.01 vs. the control group; ^#^*P* < 0.05 and ^##^*P* < 0.01 vs. the PE group.

Then, DHE staining was preformed to assess ROS production, and the results showed that the DHE expression in the PE group was higher than that in the control group, which was mitigated by HTHQ (Figure 3(a)). In addition, the SOD, CAT, and GSH levels in the PE group were significantly lower, and MDA levels were significantly higher than those in the control group (Figures 3(b)–3(e)). However, HTHQ treatment significantly upregulated SOD, CAT, and GSH levels and reduced MDA levels compared with the PE group (Figures 3(b)–3(e)).

### 3.3. HTHQ Treatment Decreased Placental Apoptosis

**Figure 4:**
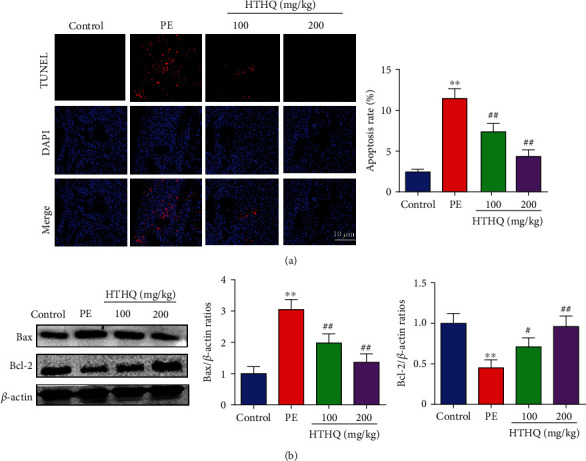
HTHQ treatment decreased placental apoptosis. (a, b) TUNEL staining was used to measure the placental apoptosis in each group (*n* = 4). ^∗∗^*P* < 0.01 vs. the control group; ^#^*P* < 0.05 and ^##^*P* < 0.01 vs. the PE group.

To further assess the protective effects of HTHQ, we used the TUNEL staining to measure the placental apoptosis. The results demonstrated that the apoptotic index in the PE group was obviously increased compared with that in the control group, while HTHQ treatment significantly decreased placental apoptosis induced by PE (Figure 4(a)). The results of western blot also showed that HTHQ treatment significantly increased Bax levels and decreased the expression of Bcl-2 in the PE group (Figure 4(b)).

### 3.4. HTHQ Treatment Attenuated Oxidative Stress and Apoptosis in HUVECs following H/R

**Figure 5:**
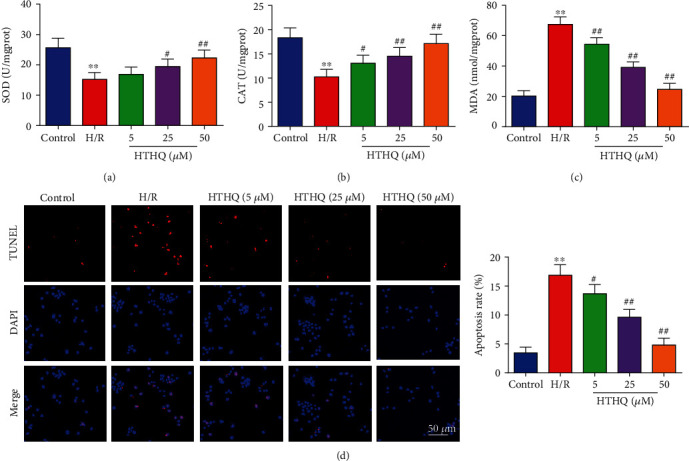
HTHQ treatment attenuated oxidative stress and apoptosis in HUVECs following H/R. The levels of SOD (a), catalase CAT (b), and MDA (c) in each group (*n* = 6). (d) Images and quantifications of TUNEL staining in each group (*n* = 6). ^∗∗^*P* < 0.01 vs. the control group; ^#^*P* < 0.05 and ^##^*P* < 0.01 vs. the H/R group.

To further assess the effects of HTHQ on oxidative stress following H/R, we measured the SOD, CAT activities, and MDA contents in HUVECs. The results revealed that the SOD and CAT activities in the H/R group were significantly lower, and MDA contents were significantly higher than those in the control group (Figures 5(a)–5(c)). However, HTHQ treatment significantly restored SOD and CAT activities and decreased MDA contents compared with the H/R group (Figures 5(a)–5(c)). In addition, HTHQ treatment significantly decreased endothelial cell apoptosis following H/R (Figure 5(d)).

### 3.5. HTHQ Activates Nrf2 Antioxidant Pathway

**Figure 6:**
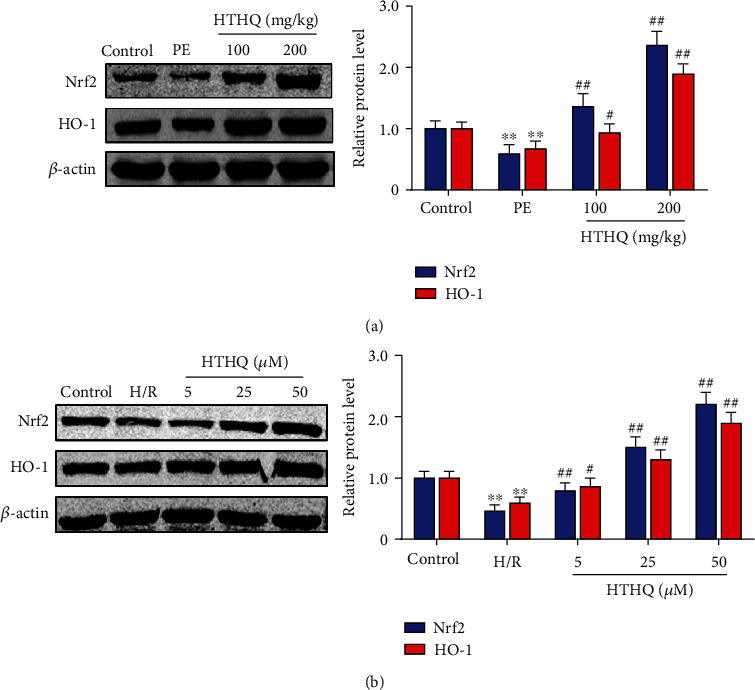
HTHQ activates Nrf2 antioxidant pathway. (a) Western blots showing the level of Nrf2 and HO-1 in mice of each group (*n* = 4). (b) Western blots showing the level of Nrf2 and HO-1 in each group (*n* = 4). ^∗∗^*P* < 0.01 vs. the control group; ^##^*P* < 0.01 vs. the H/R group.

Recent evidence has strongly suggested that HTHQ is a potent nuclear Nrf2 activator and has positive effects on oxidative stress-related diseases [16, 17]. Thus, we investigated whether HTHQ protect against PE is associated with Nrf2 antioxidant pathway. The results showed that the expression of Nrf2 and HO-1 was also significantly increased after HTHQ treatment (Figure 6(a)). In line with the data in vivo, we found that HTHQ treatment increased Nrf2 and HO-1 expression after following H/R (Figure 6(b)), indicated that HTHQ regulates PE via Nrf2 antioxidant pathway.

### 3.6. HTHQ Induces Translocation of Nrf2

**Figure 7:**
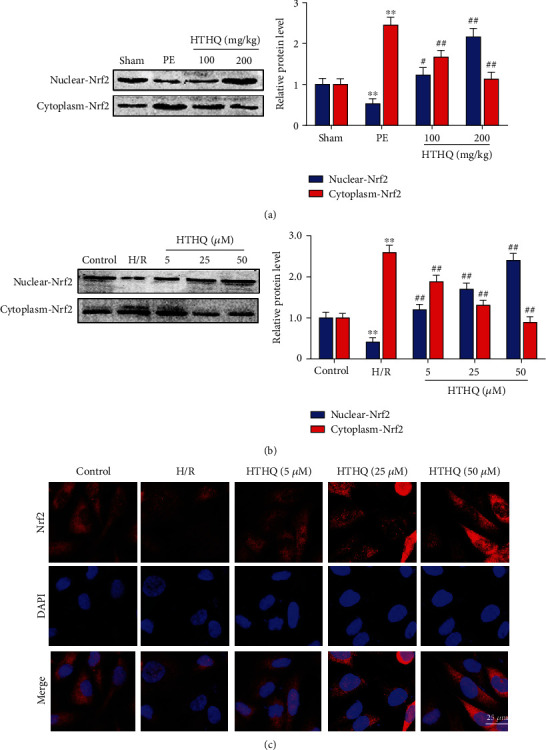
HTHQ induces translocation of Nrf2. (a) Western blots showing the level of nuclear-Nrf2 and cytoplasm-Nrf2 in mice of each group (*n* = 4). (b) Western blots showing the level of nuclear-Nrf2 and cytoplasm-Nrf2 in each group (*n* = 4). (d) Immunofluorescence staining was used to assess the translocation of Nrf2 in each group (*n* = 6). ^∗^*P* < 0.05 vs. the control group; ^∗∗^*P* < 0.01 vs. the PE or H/R group.

Then, we evaluated the effects of HTHQ on Nrf2 transcription. The results of western blots showed that the level of nuclear-Nrf2 was decreased, while the level of cytoplasm-Nrf2 was increased in the PE group compared with the sham group. In addition, HQHT treatment significantly increased the level of nuclear-Nrf2, while decreasing the cytoplasm-Nrf2 expression (Figures 7(a) and 7(b)). The results of immunofluorescence staining also showed that HTHQ has effectively induced the nuclear translocation and transcriptional activity of Nrf2 (Figure 7(c)).

### 3.7. The Protection of HTHQ Involves the Nrf2/HO-1 Pathway

**Figure 8:**
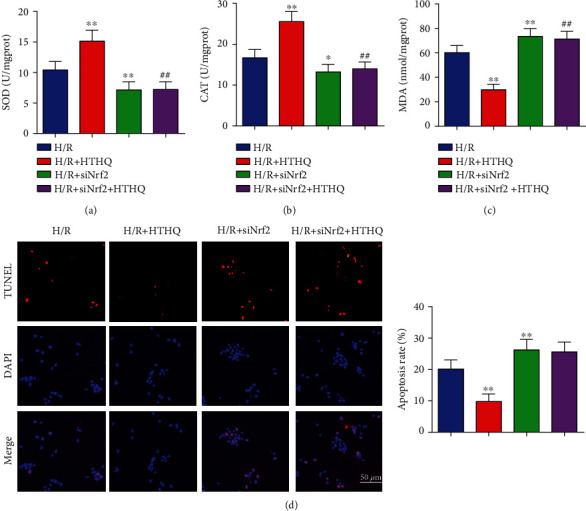
The protection of HTHQ involves the Nrf2/HO-1 pathway. The levels of SOD (a), catalase CAT (b), and MDA (c) in HUVECs after si-Nrf2 transfection (*n* = 6). (d) Images and quantifications of TUNEL staining in HUVECs after si-Nrf2 transfection (*n* = 6). ^∗^*P* < 0.05 vs. the H/R group; ^∗∗^*P* < 0.01 vs. the H/R group.

To confirm the role of Nrf2/HO-1 pathway in HTHQ-mediated protection effects, we measured the oxidative stress and cell apoptosis after si-Nrf2 transfection. The results showed that HUVECs were more vulnerable to H/R-induced oxidative stress after si-Nrf2 transfection. Meanwhile, the HTHQ-mediated protection was lost in HUVECs transfected with siNrf2 (Figures 8(a) and 8(b)). In consistent, the knockdown of Nrf2 increased the apoptosis ratio after H/R, and HTHQ treatment did not affect neuronal apoptotic in HUVECs transfected with siNrf2 (Figures 8(a) and 8(b)), indicated that the protective effects of HTHQ is involved activation of Nrf2/HO-1 pathway.

## 4. Discussion

The present study investigated the effects of HTHQ treatment on the regulation of oxidative stress and endothelial cell apoptosis in preeclamptic mice. The results showed that HTHQ treatment is able to relieve the high SBP and reduce proteinuria in a dose-dependent manner and thus improve PE. Further, we found that HTHQ treatment significantly decreased sEng, ET-1, and activin A levels and restored VEGF in preeclamptic mice. In addition, HTHQ treatment decreases oxidative stress and endothelial cell apoptosis by increasing the levels of Nrf2 and HO-1, and the HTHQ-mediated protection was lost in HUVECs transfected with siNrf2. These data provided evidence supporting HTHQ activating the Nrf2 signaling pathway, thereby protecting cells against oxidative stress injury.

ROS, the most important factor for oxidative stress, represents a family of oxygen containing molecules [28, 29]. Excessive ROS can induce placental dysfunction by suppression of placental angiogenesis, induction endothelial damage, and immune malfunction, which are suggested to be the underlie of PE development [11] [30],. In order to maintain cellular redox homeostasis, cells are equipped with antioxidant enzymes or nonenzymatic antioxidants, including SOD, CAT, and GSH, which can scavenge ROS and inhibit free radical formation [31, 32]. Previous study demonstrated that the levels of SOD, CAT, and GSH were significantly lower in women with PE than in healthy women, suggesting that the antioxidant protective capacity was decreased in women with preeclampsia [33–35]. MDA, a product of lipid peroxidation, is an important feature to estimate the degree of lipid peroxidation caused by free radical attacks [36]. Feng et al. reported that increased MDA levels could be associated with increased generation of toxic lipid peroxides and contributed to the development of PE [37].

HTHQ is a novel synthesized vitamin E derivative and has a potent antioxidant and anti-lipid-peroxidative activity. Previous research indicated that HTHQ has been considered as a promising therapeutic agent for oxidative stress-induced diseases [16] [38],. Jung et al. reported that HTHQ treatment significantly attenuated dimethylnitrosamine-induced liver fibrosis by inhibiting ROS formation and reducing lipid peroxidation. In this study, DHE staining was preformed to assess the ROS production, and the results showed that the DHE expression in the PE group was higher than that in the control group, which was mitigated by HTHQ. In addition, the SOD, CAT, and GSH activities were significantly decreased, whereas MDA level was increased in PE mice and HUVECs after H/R. Interestingly, HTHQ treatment significantly reduced the extent of oxidative stress by increasing cellular antioxidants SOD, CAT, and GSH activity and decreasing MDA level, indicated that HTHQ suppressed oxidative stress induced by PE. Nevertheless, the precise mechanisms on how HTHQ exerts protection effects still unclear.

Endothelial cells are important cell types of human placenta and contribute to the regulation of vascular tone and function. Previous research showed that endothelial damage has been proposed to be the important underlie of PE and involved in major symptoms of PE, including hypertension, proteinuria, and edema [39, 40]. Thus, improving maternal endothelial function has been proposed to be an effective therapeutic strategy to ameliorate the clinical outcomes of PE [41, 42]. In this study, we detected the role of HQHT on endothelial function. The results showed that HTHQ treatment attenuates oxidative stress in HUVECs following H/R. In addition, H/R obviously induced the apoptosis of HUVECs, while HTHQ treatment significantly decreased endothelial cell apoptosis following H/R, indicated that HTHQ could suppress endothelial dysfunction.

Nrf2 is a transcription factor and protects cells against oxidative stress, electrophiles, and carcinogenic substances [43, 44]. Under regular conditions, Nrf2 is bound to Kelch-like ECH-associated protein 1 (Keap1) in the cytosol and forms an E3 ubiquitin ligase complex, which leads to ubiquitination and proteasomal degradation of Nrf2 [45, 46]. However, when cells are exposed to oxidative and electrophilic challenges, Nrf2 is activated, released from Keap1, and binds to the ARE sequences then recruits the transcription of antioxidant defence genes [47–49]. Several evidences have shown the Nrf2 signaling pathway is a mediator of protection to counteract oxidative stress and maintain redox homeostasis in the placenta [50, 51]. Yu et al. reported that the Nrf2 and HO-1 expressions were lower in the PE placenta compared with the healthy women, and Nrf2 overexpression protected the placenta by upregulating the antioxidant genes in the murine PE model [50]. Onda et al. reported that sofalcone decreased sEng production and ameliorated placenta dysfunction by activate Nrf2 and HO-1 pathway. These researches further highlight that Nrf2 is a promising therapeutic target for the treatment of PE [51].

In the present study, we investigated the role of HTHQ treatment in Nrf2/HO-1 signaling pathway. The results showed that HTHQ treatment significantly increased the expression of Nrf2 and HO-1. In addition, HTHQ has effectively induced the nuclear translocation and transcriptional activity of Nrf2. As shown in our study, knockdown of Nrf2 could increase the level of oxidative stress and apoptosis of HUVECs under H/R. In addition, the HTHQ treatment mediated protective effect that was lost in HUVECs transfected with siNrf2. HO-1, which is regulated by Nrf2, has antioxidant and antiapoptotic activities by restoring redox homeostasis and reducing inflammation response [52, 53]. Researches showed that activation of HO-1 protects placental cells against oxidative stress injury that can improve hypertension and placental ischemia in rodents PE model [54, 55]. In this study, we also found that HTHQ treatment significantly increased the HO-1 expression in the placenta of PE. Taken together, we propose that the protective action of HTHQ is related to an antioxidant and antiapoptotic effect, and the mechanism is involved in Nrf2/HO-1 signaling pathway.

**Figure 9:**
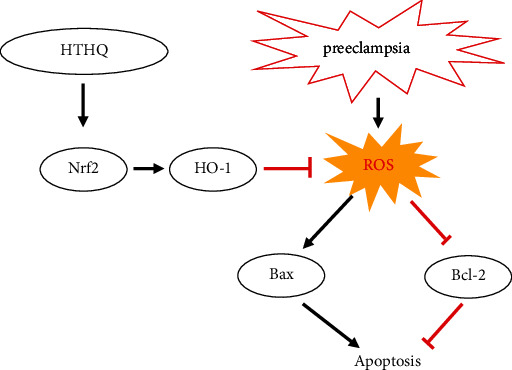
HTHQ suppresses oxidative stress and endothelial cell apoptosis and subsequent improvement of PE through activation of Nrf2/HO-1 signaling.

In summary, we have shown that HTHQ suppresses oxidative stress and endothelial cell apoptosis and subsequent improvement of PE through activation of Nrf2/HO-1 signaling (Figure 9). These findings suggest that HTHQ could be a potential pharmacological agent for PE therapy.

## Data Availability

We declare that the materials described in the manuscript, including all relevant raw data, will be freely available to any scientist wishing to use them for noncommercial purposes, without breaching participant confidentiality.
